# Analysis of Biomorphs in the Teleorman County of Romania

**DOI:** 10.3390/plants14132056

**Published:** 2025-07-04

**Authors:** Maria Magdalena Cernat Popa, Carmen Otilia Rusănescu

**Affiliations:** Department of Biotechnical Systems, Faculty of Biotechnical Systems Engineering, National University of Science and Technology Politehnica Bucharest, 060042 Bucharest, Romania; luiupuiu@yahoo.com

**Keywords:** flora, climatic conditions, bioform analysis, phytogeographic analysis, fractal analysis, transects

## Abstract

The study of flora is crucial for conserving natural resources and assessing human impact on the environment. This paper explores floristic diversity, the role of plants, and the integration of technology in botanical research. In the studied area, 462 plant species were identified. Bioform analysis revealed a predominance of *hemicryptophytes* (45.45%) and *therophytes* (26.19%), suggesting a warm climate and significant zoo-anthropogenic influences. Other bioforms were present in lower percentages. Most plant species in Teleorman County are mesophilic (39.39%) and mesoxerophilic (30.95%), indicating adaptation to moderate or slightly dry environments. Regarding temperature affinity, the majority are micromesotherms (62.98%), suited for mild thermal conditions. Soil reaction analysis showed a preference for weakly acidic neutrophilic (39.82%) and euryionic (33.76%) soils, indicating tolerance for neutral to slightly acidic pH levels. The research evaluates the structure and diversity of flora in Teleorman County and emphasizes the influence of climate factors such as humidity, temperature, and soil pH on species distribution. Using the transect method and fractal analysis, this study concludes that temperature is the dominant climatic factor shaping local biodiversity.

## 1. Introduction

The study of the flora in Teleorman County is an essential initiative in understanding and preserving the environment [1]. The county, as an administrative and geographical unit, is home to a surprising diversity of plant species, each with its own characteristics. The aim of this paper is to explore in depth the peculiarities of the flora, highlighting its importance in preserving biodiversity and supporting the sustainable development of the region [2]. The main purpose of the study of flora is to provide essential data and information for the development, implementation, and management of projects and programs for environmental protection and natural resource management. The study of flora is of major significance, as it is an integral part of the field of environmental engineering, and its main objectives are the conservation of biodiversity, since each plant species is a vital element of the local ecosystem, contributing to its balance and functioning. The registration and documentation of plant species, especially rare or threatened ones, allows the identification of important areas and the development of conservation strategies for their protection [3,4,5,6,7,8,9]. This ensures that local species do not become extinct and that genetic diversity remains available for the future. The assessment of biodiversity and environmental status through the study of flora supports environmental engineering processes through which elements of local biodiversity can be determined and the monitoring of the state of ecosystems [5,10,11,12,13,14]. This includes identifying the plant species existing in the researched area and assessing their health and diversity. The data obtained can provide insight into changes in the ecosystem and help identify environmental problems, such as habitat degradation or biodiversity loss. Contemporary urban ecosystems are ecosystems with microevolutionary genesis and processes that include a diverse spectrum of different taxonomic groups of plant and animal organisms occupying certain ecological niches of the EU [4]. The study of protected areas recognizes and incorporates the heterogeneity of landscape mosaics and involves the analysis of context and external threats [5,6,7,8,9]. The trends and relevant aspects related to the research of the flora spectrum at present are highlighted in various works that constitute a topic of interest due to the topicality of the works that is ensured by the use of working methods in the fields of botany, phytosociology, forest management, and dendroclimatology that capture a broader spectrum in terms of the dynamics of the development of flora and vegetation over time [11,12]. In the literature, it is observed that the emphasis has been placed on the methods of obtaining and processing data [11]. Research into the knowledge of plant types has significant implications and multiple benefits for environmental conservation, society, and the development of science. It makes important contributions to the understanding, protection, and sustainable use of natural resources [15,16,17,18,19]. Knowledge of the floristic composition and structure of vegetation in natural ecosystems is a key element in the conservation and sustainable management of biodiversity and the protection of species and their habitat. Cârlan mentions that vegetation monitoring requires a knowledge of the physiological processes underlying plant development and growth so that the decision-making process in the context of infrastructure management is carried out exhaustively [14].

**Figure 1:**
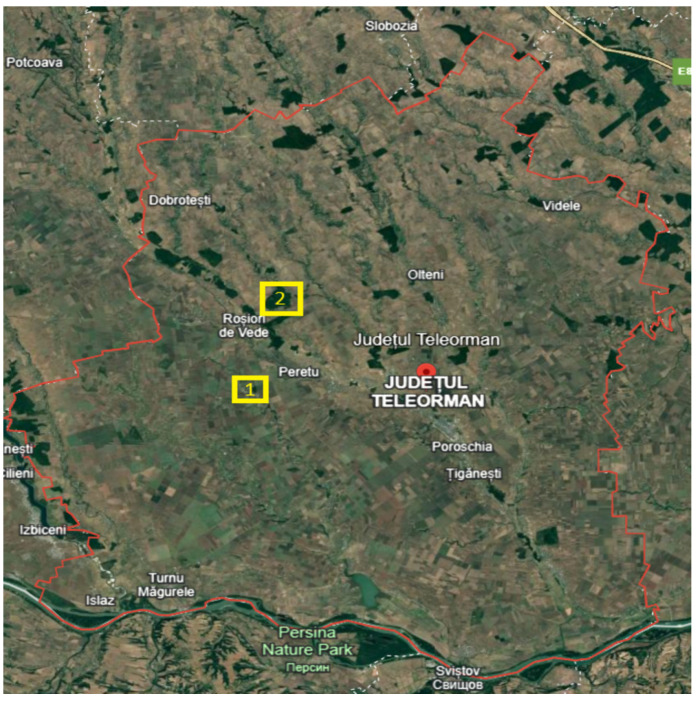
Physical–geographical map of Teleorman County, positioning of the protected areas under analysis [22]. (1) Troianul Forest. (2) Vedea Forest.

In the researched area there are protected areas such as the Troianul Forest with forest funds, and good management of this area constitutes a continuity of their existence. Forestry aims to return to the natural structures of forest ecosystems, with their self-preserving and self-regulating properties [11,12,20,21]. In the current stages of research of the flora spectra, as well as in the research area, the natural areas protected by the forest are identified. In this work, the described flora is an integral part of two natural areas: Troianul Forest and Vedere Teleorman Forest, being part of a national ensemble, the Natura 2000 Site (Figure 1) [22]. The protected areas under study are an integral part of the extensive study of the flora in the entire county, which was also carried out in unprotected areas with increased interest in the flora of these protected areas.

They are intended for the conservation of ecosystems and endangered plants, representing the most widespread tool used in conservation efforts [17,23]. The work is intended to be a lever in the scientific support of research, and in the field of biodiversity conservation, bioform analysis is carried out for this purpose. The notion of biodiversity conservation defines a protected area as “a geographically defined area designated or managed for the purpose of achieving specific conservation objectives” [3,8,15,16,17,18,19]. Studying current research, it is observed that in most protected areas there are many conflicts related to land use, especially in protected areas of flora and fauna. We consider that the incidence of their occurrence is high in developing countries [24,25,26]. Romania’s flora, an integral part of the natural framework, offers a high potential for biodiversity, which has determined that over 23% of the country’s surface is under one type or another of protection [27,28,29,30,31,32]. The research included the identification of the plant species existing in the area and their diversity with the help of establishing the points in the areas of the county by the transect method. Through the study of the flora, the local biodiversity can be evaluated, and the plant species existing in the area and their diversity can be identified. Data obtained by studying the spectrum of biological forms conclude that the geomorphology of the territory, the anthropogenic impact produced on the territory, and the category of land use can provide information about ecosystem changes and help identify environmental problems, such as habitat degradation or biodiversity loss [32,33,34,35,36]. This paper aims at a detailed analysis of the flora of Teleorman County by evaluating the diversity of species, the spectrum of bioforms, and their relationship with the main climatic factors: humidity, temperature, and soil reaction. The main objectives are to determine the structure of the flora, to identify the influence of environmental factors on the distribution of species, and to apply the transect method and fractal analysis to understand the complexity and dynamics of local ecosystems. Based on these considerations, the present paper aims to achieve the following main objectives: (1) the detailed characterization of the flora in Teleorman County, (2) the identification and analysis of the distribution of plant bioforms, and (3) the evaluation of the influence of the main climatic factors—such as humidity, temperature, and soil reaction—on the vegetation structure. To achieve these objectives, the research uses a mixed methodological approach, which combines fractal analysis, useful in determining the complexity and spatial distribution of vegetation, with the transect method, which allows the systematic collection of data along representative ecological gradients. Through this approach, this study contributes both to the scientific substantiation of the relationship between climate and the organization of local vegetation, as well as to the development of applicable solutions in the field of biodiversity conservation and sustainable management of regional ecosystems.

## 2. Results

### 2.1. Geographical and Climatic Analysis of Teleorman County

**Figure 2:**
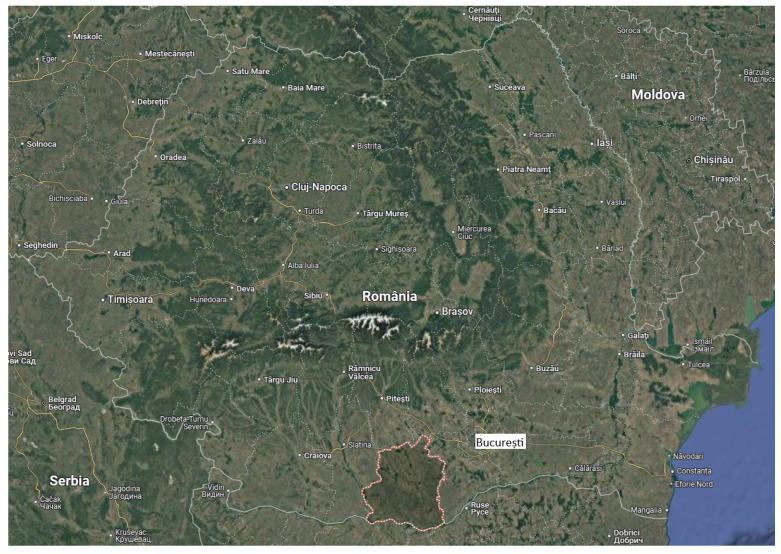
Physical–geographical map of the positioning of Teleorman County in Romania [22].

Located in the southern part of the country, Teleorman County occupies a part of the area of the Romanian Plain approximately between Olt and Vedere (Figure 2) [22]. It is intersected by the parallel of 43°37 north latitude (Zimnicea also being the southernmost peak of Romania) and by the meridian of 25° east longitude (Traian, Troianul, Roșiorii de Vede, Drăcșănei, Siliștea Nouă). Neighboring counties: Giurgiu, Dâmbovița, Argeș, and Olt. The southern boundary, formed by the Danube, corresponds to the state border with Bulgaria. From the point of view of altitude, the territory of Teleorman County stretches between 20 m in the Danube meadow and about 160–170 m in the northern part, on the border with Argeș County.

The average annual temperatures range from 11.5 °C in Turnu Magurele to 10.8 °C in Alexandria and 10.5 °C at the northern border. In July, the hottest month, the averages are higher in the south (23.4 °C in Turnu Magurele) compared to the center (22.7 °C in Alexandria) and the north of the county (about 22 °C). In January, the coldest month, temperatures drop below −3 °C in the north (−3.2 °C in Alexandria), while in the south they remain above this value (−2.3 °C in Turnu Magurele).

#### 2.1.1. Overview of the Flora of Teleorman County

The data obtained from the analyses carried out were exploited by including them in a systematic file, in which the species files were grouped by genera and families according to the current phylogenetic system of plants. The nomenclature of the species corresponds to that of the Flora of Romania. The overview of the flora of Teleorman County highlights the existence of 462 species of plants that are grouped into 69 families. In the analysis of the flora, the season, the climatic indices of humidity (U), temperature (T), and soil reaction (R), as well as the biological form and floristic element, are specified for each species.

#### 2.1.2. Bioform Analysis

**Figure 3:**
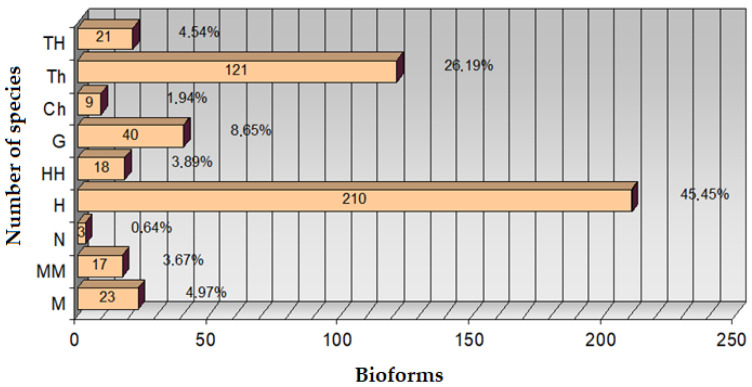
Percentage chart of bioforms.

**Figure 4:**
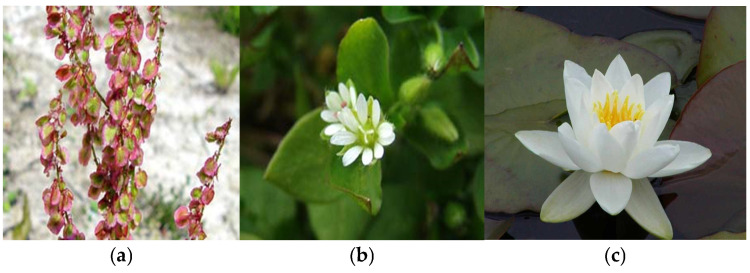
(**a**) *Rumex Sorrel*; (**b**) *Stellaria media* L.; (**c**) *Nymphaea alba* L.

By studying the spectrum of bioforms in an area, conclusions can be drawn regarding the geomorphology of the territory, the anthropogenic impact produced on the territory, and the category of land use. This is because *phanerophytes* (MM, M, N) grow in forests; *camephytes* (Ch) adapted to extreme living conditions prefer both mountaintops, rocks, or polar areas and desert areas; *hemicryptophytes* (H) grow in meadows and forests; *geophytes* (G) prefer forests; *helohydatophytes* (HH) grow only in swamps; and *therophytes* (Th) grow in deserts, steppes, in areas with dry and calm climates, on agricultural land, and in weeds. Analyzing the bioforms (Figure 3), it is observed that there is a high percentage of *hemicryptophytes* (H) of 45.45%. Dominant bioform—*hemicryptophytes* (H): number of species: 210 (45.45%). *Hemicryptophytes* represent almost half of the total species registered in the county. These plants have their renewal buds at ground level and are characteristic of temperate zones. Their dominance suggests that the flora of the county is well adapted to temperate climatic conditions, with hot summers and cold winters. Percentage of *therophyte* (Th) species, number of species: 121 out of 26.19%. *Therophytes* are annual plants that survive adverse periods in the form of seeds. A high percentage of *therophytes* indicates a climate with a short growing season or a high presence of cultivated or man-damaged land. The other types of plant species are present in a smaller percentage: *geophytes* (G)—8.65%, *camephytes* (Ch)—1.94%, *phanerophytes* (MM)—3.67%, (M)—4.97%, (N)—0.64%, and *helohydatophytes* are present in a percentage of 3.89%. The percentage scheme for the number of species of bioforms is represented in Table 1, made following the determinations. Achieving the bioform spectrum involves creating a visual representation of species diversity in a given area or habitat. The spectrum of bioform can highlight different aspects of the species community, such as distribution based on characteristics such as grouping. The spectrum of bioform is represented in Figure 4, being a valuable tool in the study and conservation of biodiversity in Teleorman County. By illustrating the diversity of species, it provides a comprehensive picture of ecological relationships and helps to make decisions in the process of managing natural resources.

**Figure 5:**
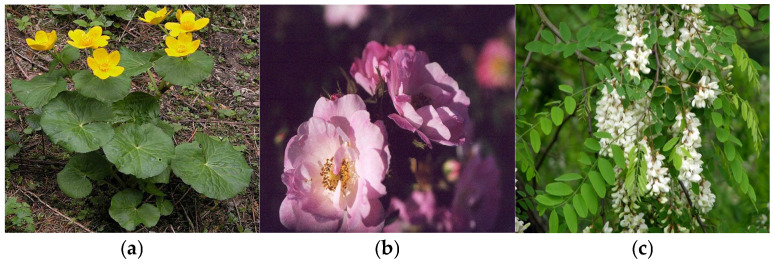
(**a**) *Caltha laeta Schott*; (**b**) *Gallic rose*; (**c**) *Robinia pseudacacia* L.

**Figure 6:**
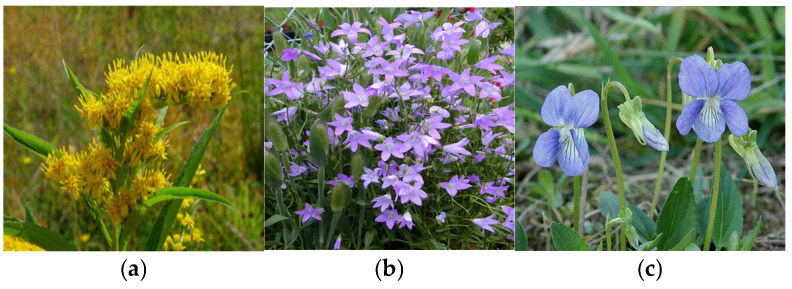
(**a**) *Solidago giant Aiton*; (**b**) *Campanula patula* L.; (**c**) *Viola canina* L.

**Figure 7:**
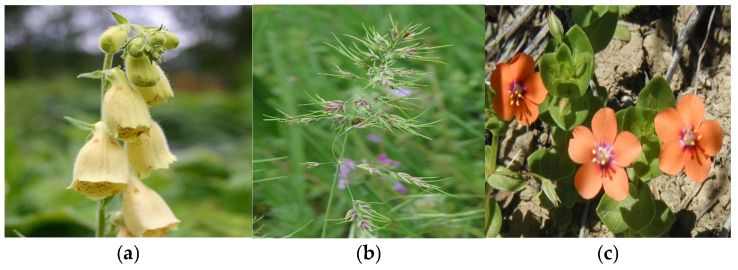
(**a**) *Digitalis grandiflora*; (**b**) *Poa bulbosa*; (**c**) *Anagallis arvensis* L.

**Figure 8:**
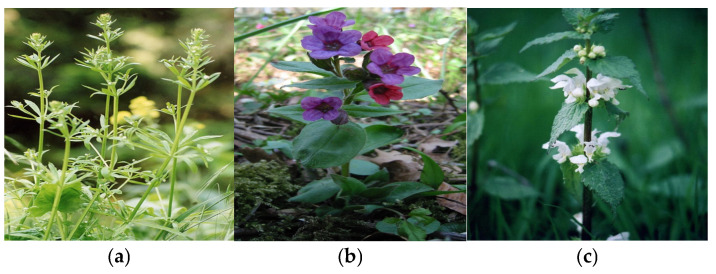
(**a**) *Galium appears* L.; (**b**) *Pulmonaria officinalis*; (**c**) *Laminium album*.

**Figure 9:**
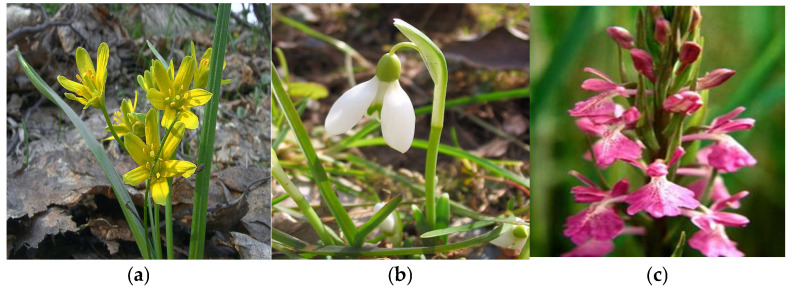
(**a**) *Gagea lutea* (L) *Ker-Gawles*; (**b**) *Galanthus nivalis*; (**c**) *Orchis laxiflora* Lam.

Identifying and classifying plant species visually [37,38,39] and according to moisture, temperature, and soil reaction indices are essential for understanding, conserving, and managing natural ecosystems. In Figure 4, Figure 5, Figure 6, Figure 7, Figure 8 and Figure 9, we made an exposition only of some vegetation species identified in the area during the study period. This practice contributes to biodiversity conservation, ecosystem health assessment, natural resource planning, and public education. It also plays an important role in adapting to climate change and restoring disturbed habitats. Through these means, we ensure that the fragile relationship between plants, the environment, and society is maintained and that natural resources are used sustainably for future generations.

**Figure 10:**
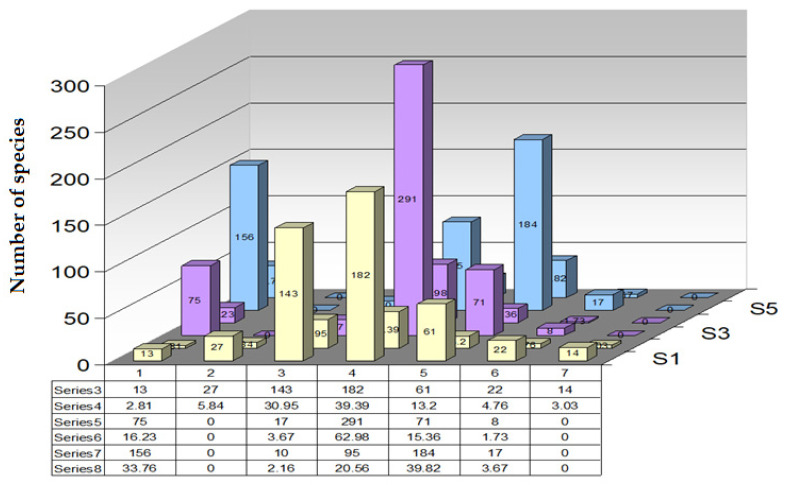
UTR spectrum of flora in Teleorman County.

Following the analyses carried out in Teleorman County on the flora, it was found that after long adaptations to environmental conditions, the current plant species have optimal growth and development between certain limits of moisture, temperature, and soil reaction. Each species has certain climatic indices for these three factors (U, T, R), marked from 1 to 6 for humidity and from 1 to 5 for temperature and soil reaction, adding the value 0 (zero). The fact that most of the flower species in Teleorman County are mesophilic species (U3–U3,5), present in a percentage of 39.39%. The mesoxerophilic species (U2–U2,5) have a fairly high share, present in a percentage of 30.95%. From the point of view of the affinity of the species existing in the flora of Teleorman County to the temperature factor, most of the species are micromesotherms (T3–T3,5), present in a percentage of 62.98%. Also, the presence of eurythermal species (T0) is noteworthy, present in a percentage of 16.23%. Following the affinities of plant species for soil reaction, it is observed that most species are low-acid neutrophils (R4–R4,5), present in a percentage of 39.82%, which means that they prefer soil with neutral to slightly acidic pH, followed by eurionic species (R0), present in a percentage of 33.76%, data presented in the graph in Figure 10.

**Figure 11:**
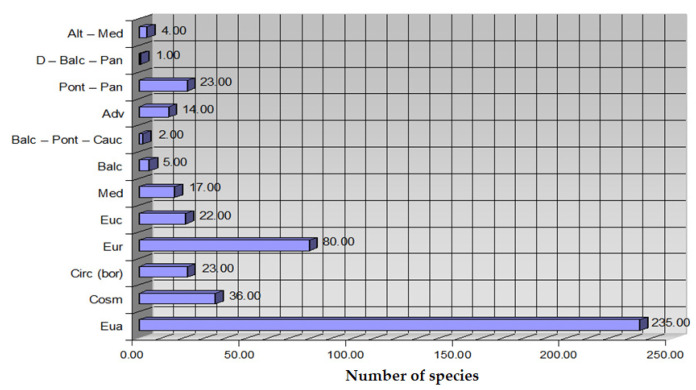
UTR spectrum of flora in Teleorman County.

For the analysis of the floristic elements, we conclude the predominance of Eurasian (*Eua*), European (*Eur*), Balkan (*Balc*), Mediterranean *(Med*), Cosmopolitan *(Cosm*), Circumpolar (*Circus*), and Central European (*Euc*) species, but also the presence of a large Pontic species (*Pontus*). The spectrum of geoelements is shown in Figure 11. Knowledge of the floristic elements is necessary, firstly, to characterize the flora of the researched region from a chorological (areological) point of view and, secondly, to make the biogeographical region of the examined area.

In Teleorman County there are a number of species of flora and fauna that have a special economic and social importance, having multiple uses in various sectors.

The species of trees and shrubs in the forests are of economic importance for the production of wood, resins, fruits, flowers, leaves, and bark, which are used for both medicinal and melliferous purposes. To perform a detailed analysis, we applied fractal data analysis [40]. In order to make a fractal graph of the relationship between climate indices (humidity—U, temperature—T, and soil reaction—R) and species distribution, we used the fractal dimension to assess whether the species distribution follows a fractal scale and graphed the relationships between various climate parameters [41].

The steps of the analysis involved simulating the data for the extant plants (462 plant species) and their distribution according to U, T, and R; calculating the fractal size using self-similarity analysis methods; and graphing to visualize the species distribution and fractal trends [42]. These analyses and the interpretation of the data help conclude the state of the environment in the studied area and indicate the influence of environmental factors on the flora in the studied area. Log-log regression is a statistical method used to identify power law relationships between variables. This is essential in fractal analysis, as many natural phenomena, including the distribution of species according to climatic factors, follow self-similar scalar patterns. In this context, we applied log-log regression to determine the fractal size of the species distribution according to environmental parameters: humidity (U), temperature (T), and soil reaction (R). The application of log-log regression using Equation (1) allows for the identification of the fractal dimension of species distribution in relation to environmental parameters (humidity, temperature, soil reaction), highlighting how floristic diversity responds in a scalable and non-uniform manner to variations in these ecological factors. This method helps quantify the influence of each factor through the exponent b, which reflects the degree of dependence of the number of species on the analyzed variable.

*Y* = *kX^b^* (1)

where:

*Y* = number of species,
*X* = climatic variable (humidity, temperature, or soil reaction),
*k* = a constant,
*^b^* = the exponential indicating the degree of fractal scaling.

Taking the logarithm on both sides of this equation, we obtain the following linear relationship:

*log*(*Y*) = *log*(*k*) + *b log*(*X*) (2)

Equation (2) shows that the relationship between the number of species and the analyzed climatic variable can be expressed linearly on a logarithmic scale, which allows for easier estimation of the coefficient *b*, indicating the influence of that variable on floristic diversity. We applied log-log regression for each of the three climatic factors:

We applied log-log regression for each of the three climatic factors.

Therefore, we performed linear regression on pairs of values (log(X), log(Y))to determine the slope b, which represents the fractal dimension of the distribution. 

We also calculated the fractal size for each parameter using the log-log regression method: fractal size for moisture (U)—0.489; fractal size for temperature (T)—0.758; and fractal size for soil reaction (R)—0.089. The fractal size *b* gives us information about the complexity and variability of species distribution in relation to each climatic factor: temperature (T) of 0.758—the distribution of species as a function of temperature is more variable and presents a more complex pattern. Species seem to be more influenced by temperature variations than by the other factors: humidity (U) of 0.489—humidity plays a role in the organization of flora, but its influence is less than that of temperature; soil reaction (R) of 0.089—almost linear, non-fractal distribution; the impact of pH on species diversity seems to be much lower compared to temperature and humidity. Temperature has the greatest impact on species diversity, having the largest fractal size (0.758). This suggests that the flora of Teleorman County is strongly influenced by temperature variations. Humidity has a moderate impact (0.489), which means that the distribution of species is partially influenced by this factor, but not as strongly as temperature. The soil reaction has a low impact (0.089), indicating that soil pH does not play a major role in floristic diversity; soil pH has a weak effect on species distribution in this region. We made graphs for the distribution of species according to humidity (U), temperature (T), and soil reaction (R).

**Figure 12:**
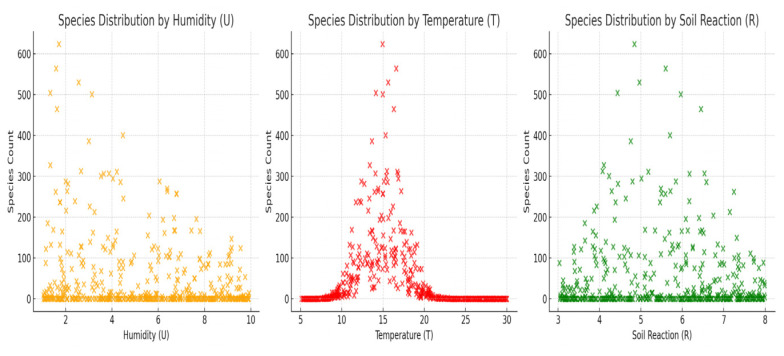
Fractal trend charts.

In the interpretation of the results, it was found that moisture (U) has a relatively small fractal size, which suggests a uniform distribution of species in relation to this parameter; temperature (T) has a larger fractal size, indicating a greater variability of species distribution depending on temperature; and soil reaction (R) has a very small fractal size, which means that the distribution of species according to soil pH is less complex, as seen in Figure 12.

For the interpretation of the data, we made the fractal trend graphs as follows:

These results suggest that temperature has the greatest impact on species diversity, having a more fractal behavior, followed by moisture, while soil reaction influences less the distribution of species in a fractal way.

**Figure 13:**
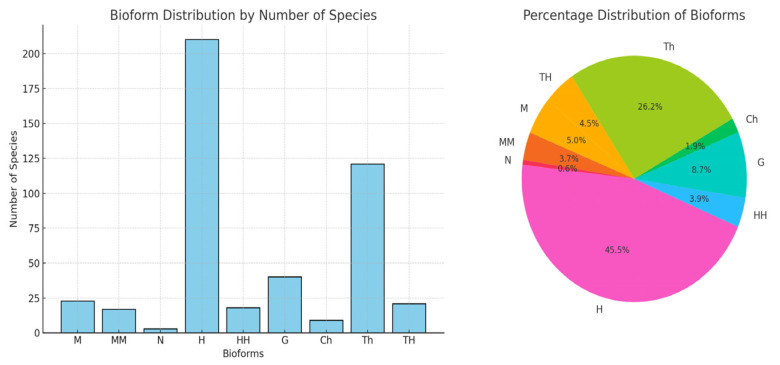
Percentage distribution of biomorphs.

For the analysis of the floristic elements, we conclude the predominance of Eurasian, European, Balkan, Mediterranean, cosmopolitan, circumpolar, and Central European species, but also a fairly large presence of Pontic species. As can be seen in the graphical representations in Figure 13, *hemicryptophytes* (H)—the dominant bioform with 45.5% of the species—are adapted to temperate zones with seasonal variations in temperature and moderate humidity. This adaptation is supported by the graph in which temperature has the greatest influence on the distribution of plants (maximum value around 14–16 °C). *Therophytes* (Th), 26.2%, indicate a habitat subject to disturbance, with short growth periods, often related to dry soils or affected by human activity. In the humidity (U) chart, the distribution is wide but smaller at the low and high extremes of humidity, suggesting adaptability to water stress. *Geophytes* (G) and *helohydathophytes* (HH), which prefer forests and swamps, are mostly found in areas with high moisture and neutral/weakly acidic soils, which is consistent with the observations in the soil reaction graph (R), although this variable has a weaker impact overall. *Phanerophytes* (M, N) and *camephytes* (Ch) have small values but occur in more stable or extreme areas. They may indicate a weaker influence of climatic factors, being often relict or adapted to specific niches. The distribution of bioforms clearly reflects the influence of climatic factors, in particular temperature, which proves to be the most important determinant of local plant biodiversity. Moisture plays a moderate role, influencing bioform groups adapted to water variations, while soil pH (soil reaction) has little impact on bioform composition. The integration of fractal analysis in the study of biodiversity is an innovative approach, as it allows the mathematical quantification of ecological complexity in a way that goes beyond classical descriptive approaches. Unlike traditional methods that assess diversity through simple inventories or standard quantitative indices, fractal analysis captures the spatial patterns of species distribution, highlighting the internal organization of ecosystems and their dynamics in relation to environmental factors. This method provides an objective tool to assess the degree of fragmentation, dispersion, or homogeneity of vegetation in a territory, which is essential in identifying sensitive areas, anticipating ecological change trends, and substantiating conservation policies. The innovation lies in the ability of fractal analysis to integrate the spatial and ecological dimensions into a unitary model, allowing an accurate diagnosis of the state of biodiversity in the region and a predictive assessment of its evolution under conditions of climatic or anthropogenic stress.

## 3. Materials and Methods

The chosen method of analysis was based on transects in different chosen areas from which plant species were subsequently sampled using systematic determinants. The advantage of the method is that instead of square squares, very elongated squares were used, and the counting of each tracked species was performed on the fly at 3 m from left to right on lengths of 500 m (2500 m^2^ in total). Going through different layers through layered sampling, we found that there are different levels of soil moisture, temperature, and reactivity. The research methodology was chosen by the author of this study based on the scientific validity of the Ellenberg ecological values and fractal analysis, internationally recognized for the evaluation of the distribution of vegetation in relation to environmental factors. This study was applied in areas of Teleorman County in Romania, such as Troianul Forest, Vedea Forest, Suhaia Pond, pasture areas, and urban areas. The county is a lowland region in the south of the country, characterized by a temperate continental climate and significant anthropogenic influences. The research was carried out between April and August 2022, covering the main growing season to allow for full observation and recording of floristic diversity. These study methods were chosen because of their ability to provide a detailed, quantitative, and comparable assessment of the distribution of species in relation to major environmental factors (temperature, humidity, and soil reaction).

**Figure 14:**
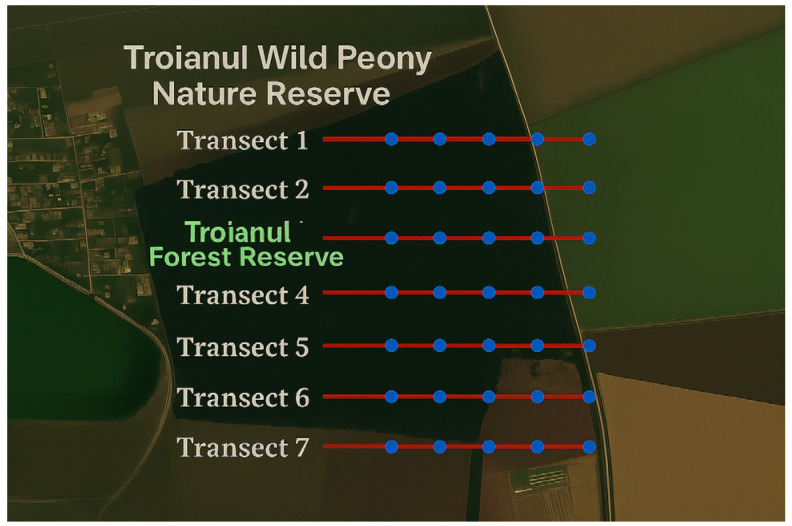
Scheme for the application of transects in the protected area—Troianul Forest Reserve.

**Figure 15:**
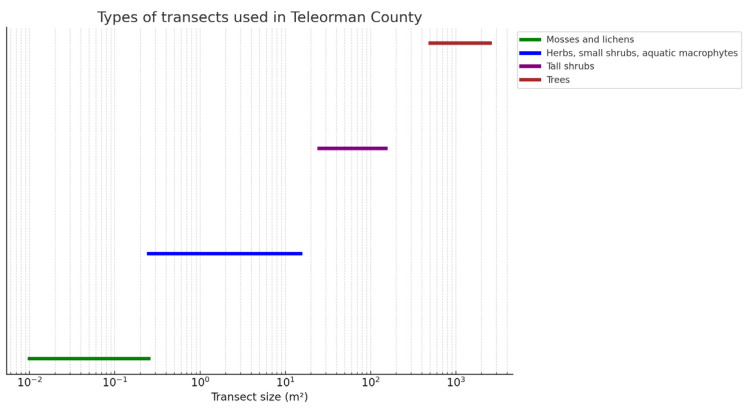
Diagram of transects used in Teleorman County.

Figure 14 shows the positioning of the transects that were placed along the main lines in one of the studied areas, the Troianul Forest Reserve, proceeding exactly with the other areas that are part of the research. The technique used to determine the plant community for mosses and lichens consisted of changing the dimensions used from transects of 0.01–0.25 m^2^ to transects of 0.25–15 m^2^ for grasses, small shrubs, and aquatic macrophytes; 25–150 m^2^ for tall shrubs; and 500–2500 m^2^ for trees, as shown in Figure 15. The sketch and analysis of the flora in Teleorman County were made based on field observations made in the summer and autumn of 2022.

The transect method was used for the systematic investigation of the flora in Teleorman County by establishing straight lines (transects) drawn on the ground in various types of habitats (forests, meadow areas, agricultural lands, meadows, etc.), along which direct observations and records of the plant species encountered were made. These transects were distributed in such a way as to reflect local ecological variations (soil, exposure, altitude, and humidity) and to allow a balanced sampling of vegetation. The data collected on the transect segments were then analyzed from the point of view of spatial distribution and correlated with fractal analysis, which provided a quantitative interpretation of the degree of aggregation, dispersion, or fragmentation of the identified species. The correlation between the two methods is innovative because transects provide a standardized empirical framework for observations, while fractal analysis turns these data into numerical indicators of ecological complexity. This combined approach allows not only to describe the floristic composition but also to highlight the self-organized structures and natural adaptation strategies of plants according to environmental conditions. The novelty of this method lies in the integrated application of two complementary techniques—one specific to environmental engineering and one mathematical—to obtain a deeper and more rigorous picture of the relationships between flora and environmental factors in a specific territory, contributing to the advancement of research in the field of environmental engineering and biodiversity conservation.

In order to validate the identification of the species, direct field observations were used, supplemented by taxonomic determinations made with the help of reference floristic works, such as “Flora of Romania” and specialized atlases. The correct identification of taxa was supported by consulting updated botanical works and, where necessary, by checking the nomenclature in international databases such as Euro + Med PlantBase and The Plant List. For measurements, such as the analysis of ecological affinities (humidity, temperature, soil reaction), standardized ecological scales (e.g., Ellenberg) and correlations with the climatic and edaphic data available at the regional level were applied. The values obtained were compared with the specialized literature to ensure the consistency and ecological validity of the interpretations. This double validation—botanical and environmental engineering—allowed the solid foundation of subsequent analyses on bioforms, distribution, and relationships with environmental factors.

The application of the Ellenberg ecological scale in the field involved the association of each identified species with the estimated ecological values for the main environmental variables: humidity (F), temperature (T), soil reaction/pH (R), nutrient content (N), and luminosity (L). These values are not measured directly in the field but are assigned on the basis of standardized tables developed by Heinz Ellenberg and subsequently supplemented by other European authors, in which each plant species is classified according to its ecological preferences. In the field, we collected the plants, made the list of plant species present on a certain transect, and then, in the analysis, assigned to each plant species the values of the corresponding Ellenberg scale. The average of these values for each factor allows the indirect estimation of the prevailing environmental conditions in that area. Thus, the method provided a quick and efficient assessment of habitats based on floral composition, without the need for direct measurement of environmental factors.

Steps to obtain ecological values based on the Ellenberg scale are as follows:

1. Establishment of transects and floristic collection

Transects were drawn in the field (Figure 14) in various habitat types of representative of Teleorman County (e.g., forest, meadow, marginal agricultural lands). Direct observations were made on each transect, and the plant species present were recorded.

2. Identification of plant taxa

The species were determined based on morphological characteristics, using specialized flora works, such as “Flora of Romania”, and regional botanical atlases. In ambiguous cases, additional taxonomic determinations were made by consulting the literature and databases (Euro + Med PlantBase, The Plant List).

3. Associating each species with Ellenberg ecological values

For each species identified, the corresponding ecological values were extracted from the standardized Ellenberg tables for the following factors:

F—humidity;

T—temperature;

R—soil reaction (pH).

4. Calculation of averages on each environmental factor

After assigning the values of each species, the arithmetic means for each factor (F, T, R) were calculated, weighted with the frequency of occurrence of the species on the transects. These average values indirectly reflect the environmental conditions in the studied areas.

5. Interpreting the results

The mean values obtained were then classified in the descriptive classes (e.g., mesophyll, mesoxerophil, micromesotherm, weak acid–neutrophil, etc.), according to the standard interpretations in the literature. This step allowed the drafting of ecological conclusions on moisture, temperature, and soil reaction in the analyzed areas.

The application of fractal analysis in this study provides an innovative tool for investigating plant biodiversity through its ability to reflect the complexity and spatial organization of species distribution. This method allows the mathematical quantification of the degree of fragmentation, density, and dispersion of vegetation, thus being able to capture how environmental factors—such as temperature, humidity, and soil pH—directly influence the organization of ecosystems. The calculated fractal size highlights the self-organized structure of vegetation, providing an overview of the ecological relationships between plants and their habitat. In addition, the transect method, used to collect floristic data in the field, ensures systematic sampling of different types of habitats, contributing to the identification of the real distribution of species in relation to ecological variability. The identification of the species was carried out through direct observations and consultation of specialized regional flowers, corroborated taxonomic checks to ensure the accuracy of the data. This integrative approach supports not only the precision of the analysis but also the originality of the scientific approach, offering a complex and rigorous perspective on the local flora and its relations with the environment.

Calculation of Ellenberg ecological values:

To assess the relationship between species and local ecological conditions, the Ellenberg ecological values, recognized at the European level for characterizing plant preferences over environmental factors, were applied. Thus, each identified species was associated with the corresponding numerical values for moisture (F), temperature (T), and soil reaction (R), based on standardized tables in the literature. Subsequently, the weighted average of each ecological factor was calculated, depending on the frequency of occurrence of species on transects. The applied formula is as follows:

(3) $$\bar{X}=\frac{\sum_{i=1}^{n}(X_{i}\cdot f_{i})}{\sum_{i=1}^{n}f_{i}}$$

where:

*Xi =* Ellenberg value for the species,

*f_i_ =* the recurrence of the occurrence of the species on transects,

*n* = total number of species,

$\bar{X}$ = weighted average of the analyzed factor.

The following 5 dominant species have been identified on a transect:

1. *Plantago lanceolata*—F = 5, T = 6, R = 5, frequency: 20 occurrences;
2. *Taraxacum officinale*—F = 6, T = 6, R = 6, frequency: 15 occurrences;
3. *Achillea millefolium*—F = 4, T = 5, R = 6, frequency: 10 occurrences;
4. *Poa pratensis*—F = 6, T = 6, R = 6, frequency: 8 occurrences;
5. *Stellaria media*—F = 5, T = 7, R = 7, frequency: 7 occurrences.

Moisture calculation (F):

(4) $$\bar{F}=\frac{\left(5\cdot 20)+(6\cdot 15)+(4\cdot 10)+(6\cdot 8)+(5\cdot 7\right)}{20+15+10+8+7}=\frac{100+90+40+48+35}{60}=\frac{313}{60}\approx 5.22$$

For the temperature, the same type of calculation and the soil reaction were applied. The resulting mean for all transects in the county was humidity (F) ≈ 5.08, temperature (T) ≈ 6.08, and soil reaction (R) ≈ 5.92.

These aggregate values confirm the predominance of a mesothermic climate, with moderate humidity and weakly acidic–neutral soils. Soil reaction bioform analysis is a technique used to assess and understand the distribution of plant species in a given ecosystem. This method is based on observing plant species’ preferences for certain soil characteristics, such as pH (acidity or alkalinity), chemical composition, texture, or other properties. The basic steps taken for the analysis of soil reaction bioforms are collecting soil data from the selected transects by collecting samples, recording detailed information about each soil sample, including pH, nutrient content, and relevant characteristics, identifying plant species, noting transects and identified species, classifying species according to soil preferences, analyzing data with the help of statistical analysis and making diagrams, and interpreting results. Full sets of buffers were used, which included solutions for calibration at multiple pH values, such as 4.01, 7.00, and 10.01. These kits provided a wider range of solutions to check and calibrate the pH meter and accurately identify soil reaction as well as the nutritional support characteristics of plants [43]. For the classification of bioforms according to the moisture index, the plants were weighed before and after drying, going through the following steps: collecting the plants from the transects and weighing them carefully to record their initial weight. Drying the plants at a constant temperature (at 70–80 °C) for several hours to remove all the water, weighing the dried plants again, and calculating the difference in weight to determine the water content. The classification of bioforms according to the temperature index was performed by using existing climate data, such as monthly or annual average temperatures, to identify temperature patterns in the study area. The correlation of climate data with the distribution and behavior of plant species was arranged to determine their thermal preferences. In the analysis of the climatic indices, the Annual Average Temperature Index (MAT), the Annual Thermal Variation Index (AAT), or the Seasonal Temperature Index (TSI) were taken into account to assess the thermal environment of the studied area and the identified plant species. The integration of the three methods—transects, fractal analysis, and the Ellenberg scale [44,45]—allowed a complementary and interdisciplinary approach, providing a complex ecological diagnosis that correlates the spatial distribution of vegetation with ecological determinants.

The floral ecosystem in Teleorman is strongly influenced by the thermal factor, which explains the predominance of bioforms adapted to heat stress (hemicryptophytes, therophytes) and the irregular distribution of species, typical for ecologically changing areas. The vulnerability to global warming → flora has a high sensitivity to temperature variations. The results obtained in Teleorman County—where hemicryptophytes (45.45%) and therophytes (26.19%) predominate, and the most frequent Ellenberg values indicate a moderate mesothermic climate (F ≈ 5.08; T ≈ 6.08; R ≈ 5.92)—partially align with, but also contrast with, other research conducted in adjacent or ecologically different regions:

To highlight the ecological specificity of Teleorman County, the results obtained in this study—particularly the average Ellenberg values (F = 5.08; T = 6.08; R = 5.92) and the structure of life forms (*hemicryptophytes*—45.45%; *therophytes*—26.19%)—were compared with real data published from other regions of Romania: the Oltenia Plain, the Getic Subcarpathians, and the Danube Delta.

In the case of the Oltenia Plain, the study conducted by Vlăduț, Nikolova, and Licurici (2017) [46] reveals an increased influence of intensive agriculture and a slightly higher pluviometric regime (F ≈ 5.9), which favors a higher proportion of *therophytes* (over 35%) and indicates significant anthropogenic pressure on natural ecosystems. Temperatures are similar to those recorded in Teleorman (T ≈ 6.2), although the soils are slightly more acidic.

In the Getic Subcarpathians, according to the research by Neniu and Vlăduț (2017) [46], the vegetation is adapted to a significantly more humid (F ≈ 6.7) and cooler climate (T ≈ 5.5), which supports the development of *phanerophytes* and *chamaephytes*—life forms typical of mountainous and moist areas. This type of vegetation is also tolerant of acidic soils, in contrast to the eurionic flora of Teleorman.

In the Danube Delta, the study by Schneider-Binder (2018) [47] on aquatic macrophyte communities in the Gorgova–Isac–Uzlina area reveals a dominant presence of hydrophytes, with very high Ellenberg values for moisture (F ≈ 7.5) and a slightly alkaline soil reaction (R ≈ 6.5). These data reflect edaphic and climatic conditions that are entirely different from those found in the southern lowlands of Romania.

**Figure 16:**
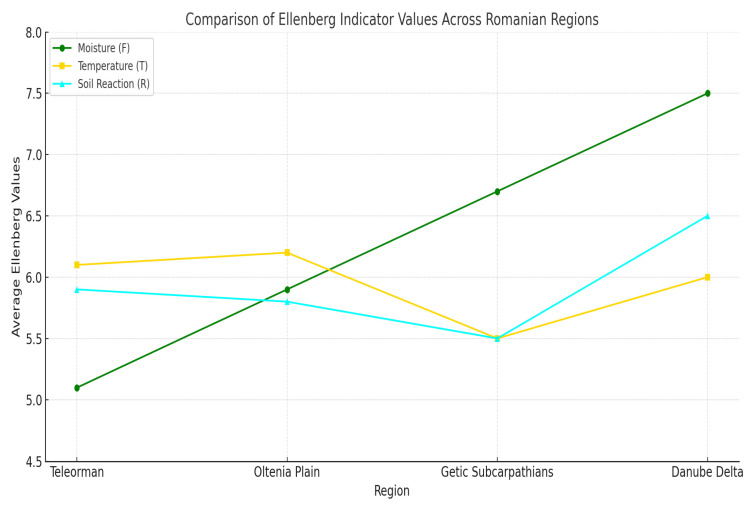
Comparative diagram regarding the Ellenberg values obtained from other regions of Romania: Oltenia Plain, Getic Subcarpathians, Danube Delta, and Teleorman County.

Therefore, the comparative analysis confirms that the flora of Teleorman County develops within an intermediate ecological context, characterized by a mesothermal climate, moderate humidity, and slightly acidic to neutral soils (Figure 16). This ecological configuration supports an adaptive biodiversity, where perennial and annual species coexist and are capable of withstanding seasonal thermal stress and moderate anthropogenic influence. Moreover, the integrated application of the fractal–transect method and Ellenberg values within a unified methodological framework gives this study significant scientific value, complementing the national biodiversity map and providing useful data for conservation strategies in the southern lowland regions of Romania.

The forest of Teleorman County is distinguished by a unique ecological configuration at the national level, the result of the interaction between a mesothermic climate, moderate humidity, and soils with a weak acid–neutral reaction. The present study, through the combined application of fractal analysis and the transect method, highlights not only a high floristic diversity with 462 identified species but also spatial tendencies of self-organization of vegetation clearly correlated with local climatic variables. This integrated approach is rarely found in the Romanian literature, and the calculated fractal dimension offers an innovative perspective on the relationship between bioforms and the environment. Compared to other studied regions (e.g., Getic Subcarpathians, Danube Delta), Teleorman is distinguished by its ecological balance and high adaptability of species, which makes this study a valuable methodological landmark and an original contribution to the assessment of biodiversity in lowland areas subject to climatic and anthropogenic pressures.

### 3.1. International Connections and Comparative Relevance

The results obtained by using the Ellenberg ecological values allow a direct correlation with studies carried out in other European regions. In Germany and Poland, the average values for moisture are between 6 and 6.5, the temperature is between 5.8 and 6.2, and the soil reaction is around 6–6.3 [48]. In comparison, Teleorman falls in humidity (F = 5.08) as a moderately dry region, with a temperature (T = 6.08) close to the Central European values and a soil with a weak acid–neutral reaction (R = 5.92), reflecting a flora adapted to a mesothermic and balanced climate. French studies on lowland vegetation indicate values similar to those in Teleorman, which reinforces the comparative relevance of the research.

The international importance of this study lies in the integrated application of fractal analysis and Ellenberg values in an area of the Eastern European plain, rarely studied at this level of detail. This approach provides replicable data in a global context and contributes to the completion of European floristic databases, with the potential to become a methodological benchmark in the monitoring of plant biodiversity on a continental scale.

Numerous international research studies support the application of these methodologies in the assessment of plant biodiversity. Tichý, L., Chytrý, M., et al. (2022) [49] used Ellenberg indicators for the analysis of plant communities in Germany’s temperate zones, emphasizing the response of species to climatic variations. Grabherr, G., Gottfried, M., and Pauli, H. (2010) [50] integrated Ellenberg values into studies on the effects of climate change on alpine vegetation in Austria. In Poland, Szymura, M. and Szymura, T. H. (2016) [51] demonstrated the validity of this method for investigating forest and wetland flora, obtaining data comparable to those in southern Romania. Perring, M. P., Bernhardt-Römermann, M., and Baeten, L. (2018) [52] applied fractal analysis in fragmented agricultural landscapes in France, highlighting the irregular pattern of species distribution in relation to anthropogenic stressors.

An integrated application of fractal analysis and Ellenberg values in an Eastern European plain area is rarely studied at this level of detail. This approach provides replicable data in a global context and contributes to the completion of European floristic databases, with the potential to become a methodological benchmark in the monitoring of plant biodiversity on a continental scale.

### 3.2. Comparison Between the Teleorman Study and International Research

This study carried out in Teleorman County is methodologically and thematically aligned with recent floristic research in Central and Western Europe. Similar to the analysis by Tichý et al. (2022) [49], from Germany, who used Ellenberg values to assess plant communities’ reactions to climate change, the Teleorman study applies the same indicators to determine the influence of soil moisture, temperature, and pH on flora. The values obtained in Teleorman (F = 5.08; T = 6.08; R = 5.92) are placed in the vicinity of those reported by Tichý (F ≈ 6.2; T ≈ 5.8; R ≈ 6.3), which demonstrates similarities in vegetation structure between German mesothermic zones and southern Romania.

In Austria, Grabherr et al. (2010) [50] highlighted the adaptability of the Ellenberg indicators to alpine ecosystems, and in Poland, Szymura and Szymura (2016) [51] validated this method for the assessment of forest and riparian flora. However, the Teleorman study makes a unique contribution, as it applies the same methods in an Eastern European lowland region, where the flora is subject to different edaphic and climatic conditions—in particular a lower rainfall regime and a stronger anthropogenic influence.

Moreover, the application of fractal analysis in plant landscapes in France by Perring et al. (2018) [52] allowed the detection of irregular patterns of species distribution in relation to anthropogenic stress. The Teleorman study takes this approach and adapts it to the lowland environment, demonstrating that temperature is the main factor that determines the complexity and structure of the local vegetation. Thus, the integration of this method in the analysis of the Romanian flora represents a rare and valuable methodological contribution, comparable to the most recent international research trends.

The methodology used combines fractal analysis with Ellenberg values in an Eastern European context poorly represented in the global literature. Detailed floristic traces for 462 species help complement global databases on species distribution and ecological conditions, which are valuable for climate and conservation research at a continental scale. The integration of the fractal dimension for the description of the spatial organization of vegetation is less used in Southeast Europe, and its application on real data in this study can serve as a replicable model for other temperate ecosystems. The results obtained allow international comparability by using a standardized methodology (Ellenberg values), allowing correlation with studies from Germany, Austria, Poland, or France. The conclusions highlight that temperature is the main determinant of the structure of bioforms—a valuable observation in the context of current climate change, providing benchmarks for predictive models in other regions with similar characteristics.

## 4. Conclusions

The analysis of the flora in Teleorman County showed a predominance of *hemicryptophytes* (45.45%) and *therophytes* (26.19%), indicating a flora adapted to a warm climate and significantly influenced by zoo-anthropic factors. Other types of bioforms, such as *geophytes*, *camephytes*, *phanerophytes,* and *helohydatophytes*, are present in smaller percentages. From the ecological perspective, most species are mesophilic (39.39%) and mesoxerophilic (30.95%), and in relation to temperature, micromesothermic (62.98%) and eurythermal (16.23%) species predominate. As for the soil reaction, the species prefer weakly acidic–neutral soils (39.82%) and have a good tolerance to pH variations (eurionic—33.76%).

The results of the fractal analysis demonstrated that temperature is the main factor influencing the diversity and distribution of species, having a high ecological complexity. Moisture has a medium impact, and soil reaction has a low effect, with a more uniform distribution of species. These observations confirm that the biological structure of the flora of Teleorman is majorly determined by climatic conditions, especially temperature. The balance between perennial bioforms and annual species reflects a complex adaptation to the variability of the local environment, providing a clear picture of the relationship between vegetation and ecological factors in a representative regional context.

The ecological diagnosis of the southern plain area of Teleorman County, carried out by integrating the transect method, fractal analysis, and the Ellenberg scale, highlights an ecosystem characterized by moderately stable vegetation but with a marked spatial fragmentation depending on heat stress. The transects allowed the precise identification of species distribution and habitat variability, providing a solid basis for ecological interpretations. Fractal analysis showed that temperature is the main determinant of vegetation complexity (Df = 0.758), while moisture (Df = 0.489) and soil reaction (Df = 0.089) had less influence. The Ellenberg scale added ecological significance to these results, indicating the predominance of mesophilic and micromesothermic species, adapted to moderate humidity and temperature conditions, but also a significant presence of hemicryptophytes and therophytes, suggesting an environment with seasonal stress and anthropogenic influence. Overall, the flora of this region is tolerant to edaphic variations but sensitive to climate change, which makes it vulnerable in the context of global warming while providing a relevant model for assessing plant biodiversity in ecological transition areas in Southeast Europe.

## Figures and Tables

**Table 1:** Percentage scheme of bioforms.

Bioform	Number of Species	[%]
M	23	4.97
MM	17	3.67
N	3	0.64
H	210	45.45
HH	18	3.89
G	40	8.65
Ch	9	1.94
Th	121	26.19
TH	21	4.54
Total	462	100.00

## Data Availability

Data are contained within the article.
